# Supplementary material for: Social capital, depressive symptomatology, and frailty among older adults in the western areas of China
Source: PLoS One. 2023 Oct 3;18(10):e0292236. doi: 10.1371/journal.pone.0292236 (PMC10547179; doi:10.1371/journal.pone.0292236)
Supplement: Attachment — Submitted filename: Response to Reviewers.docx [file pone.0292236.s002.docx]

**Response letter**

Dear editor:

We are very glad to submit our revision titled “Social capital, depressive symptomatology, and frailty among older adults in the Western areas of China” to your journal. We amended the funding statement as follows: this work was supported by the National Natural Science Foundation of China (grant number 81860599), and addressed all the comments from the reviewers point by point.

Your sincerely

Zhizhong Wang, Ph.D.

**Comments from the Editor**

1.Please ensure that your manuscript meets PLOS ONE's style requirements, including those for file naming.

**Response:** Yes, we have revised the whole article along with PLOS ONE's style requirements.

2. We noticed you have some minor occurrence of overlapping text with the following previous publication(s), which needs to be addressed:

[https://www.longdom.org/open-access-pdfs/a-confusing-general-term-frailty-should-be-organized-in-relation-with-frieds-criteria-for-frailty-locomotive-syndrome-mu.pdf](https://www.longdom.org/open-access-pdfs/a-confusing-general-term-frailty-should-be-organized-in-relation-with-frieds-criteria-for-frailty-locomotive-syndrome-mu.pdf)
[https://www.researchgate.net/publication/310021666_Social_capital_interventions_targeting_older_people_and_their_impact_on_health_A_systematic_review](https://www.researchgate.net/publication/310021666_Social_capital_interventions_targeting_older_people_and_their_impact_on_health_A_systematic_review)
[https://bmcgeriatr.biomedcentral.com/articles/10.1186/s12877-022-02815-z](https://bmcgeriatr.biomedcentral.com/articles/10.1186/s12877-022-02815-z)

In your revision ensure you cite all your sources (including your own works), and quote or rephrase any duplicated text outside the methods section. Further consideration is dependent on these concerns being addressed.

**Response:** As the reviewers suggested, we have cited the previous studies, and re-phrased the sentences through whole manuscript. All the changes have highlight in red color.

3. We note that the grant information you provided in the ‘Funding Information’ and ‘Financial Disclosure’ sections do not match.

**Response:** As we described in the funding part, “this work was supported by the National Natural Science Foundation of China (grant number 81860599)”. The funder had no role in study design, data collection and analysis, decision to publish, or preparation of the manuscript.

4.Thank you for stating the following financial disclosure:
"The authors received no specific funding for this work."

At this time, please address the following queries:

a) Please clarify the sources of funding (financial or material support) for your study. List the grants or organizations that supported your study, including funding received from your institution.
b) State what role the funders took in the study. If the funders had no role in your study, please state: “The funders had no role in study design, data collection and analysis, decision to publish, or preparation of the manuscript.”
c) If any authors received a salary from any of your funders, please state which authors and which funders.
d) If you did not receive any funding for this study, please state: “The authors received no specific funding for this work.”

Please include your amended statements within your cover letter; we will change the online submission form on your behalf.

**Response:** we amended the funding statement within our cover letter as well as in the main text.

5. We are unable to open your Supporting Information file [database.sav]. Please kindly revise as necessary and re-upload.

**Response:** Now we provided the supporting Information file as EXCEL format.

6. Please include captions for your Supporting Information files at the end of your manuscript, and update any in-text citations to match accordingly. Please see our Supporting Information guidelines for more

**Response:** We added a title named Supplementary Table 1. original data at the top of our supplementary file (in the first row), and cited it in body manuscript, described it as “Thus, 2,591 subjects (Supplementary Table 1) were included in the final analysis via a face-to-face structural questionnaire survey ”, can be seen in Participants part, line 100-102.

**Reviewer’s Comments to the Author**
**Reviewer #1**

1. Pay attention to the tense and grammar of the article in writing.

**Response:** We have checked the grammar error over the whole manuscript by a English speaker. And all the changes have highlight in red color.

2. In Table 2, "After controlling for covariates, SC was negatively correlated with frailty (r=0.07, P=0. 001) and SC was also negatively correlated with depressive symptoms (r=-0.08, P<0.001)", the correlation coefficient seems to be too small for the authors to re-verify the statistical significance.

**Response:** We have checked the data and re-runed the analysis again, the statistical significance persist, we agree the small effect size, and discussed this in the maintext.

3. Line 190, r = -0.07 instead of 0.07.

**Response:** We have revised it and marked in red.

4. The discussion section authors should focus on how depression plays a moderating role in social capital and debilitation.

**Response:** We supplement the description of moderating role of depression in social capital and debilitation, which can be seen in the last paragraph of discussion part, and marked in red.

**Reviewer #2**

1. Abstract: Line 44~45: ” SC was identified as a protective predictor of frailty in Chinese old(er) adults in this study…” The authors need to be very careful of making this conclusion because it just shows association but not causality. Does this conclusion overreach?
**Response:** we revised the description as “SC was identified as a possible protective predictor of frailty in……”.
2. Participants: Line 100: Sample numbers were inconsistent. (2681-90 (42+22+26) = 2591)
**Response:** We revised it and marked in red, Totally, 2,681 community residents enrolled and finished the face to face survey, of them, 64 were excluded due to the missing value of frailty scale (n=42), and PHQ-9 (n= 22), and 26 aged less then 60 were excluded. Thus, 2,591 subjects (Supplementary Table 1) were included in the final analysis.

3.Results: Line 199~203: Mediation effect of depression on the relationship of SC and frailty was not explained clearly enough. Is the statistical analysis adequate? Is there any possible confounder?
**Response:** we revised the description of mediation effect of depression on the relationship of SC and frailty, now described as “after controlling for covariates, there is a significant mediation effect of depression on the relationship between SC and frailty. The total effect SC on frailty was significant (total effect, β = -0.011; 95% CI = -0.017,– 0.005; P = 0.007). The estimated β (95% CIs and P value) of a significant indirect effect mediated by depression was -0.004 (95% CI = -1.006,–0.002 and P = 0.039). The mediation effect explained 36.4% (-0.004/-0.011) of the total variance”. The statistical analysis is adequate, and we controlled the possible confounders includes age, gender, marital status, educational attainment, sleep quality, health behaviors and physical health as described in methods.

4. “Older adults” is better wording than “old adults”, it should be constantly the same all through the paper.
**Response:** we changed “old adults” into “older adults” among the whole article.

5. English editing and grammatical adjustment are required.
**Response:** We have checked the grammar error over the whole manuscript by a English speaker. And all the changes have highlight in red color.

6. Please rearrange Table 1 and Table 3 to be shorter and neater.

**Response:** we have re-arranged the Table 1 and Table 3, now they are shorter and cleaner.
